# Bio-Inspired Swarm Confrontation Algorithm for Complex Hilly Terrains

**DOI:** 10.3390/biomimetics10050257

**Published:** 2025-04-22

**Authors:** He Cai, Fu Ma, Ruifeng Ni, Weiyuan Xu, Huanli Gao

**Affiliations:** 1 School of Automation Science and Engineering, South China University of Technology, Guangzhou 510641, China; caihe@scut.edu.cn (H.C.); 202321016838@mail.scut.edu.cn (R.N.);; 2 Key Laboratory of Autonomous Systems and Networked Control, Ministry of Education, Guangzhou 510641, China; 3 Guangdong Engineering Technology Research Center of Unmanned Aerial Vehicle Systems, Guangzhou 510641, China

**Keywords:** bio-inspired algorithm, swarm confrontation algorithm, hilly confrontation scenario, swarm animal behaviors

## Abstract

This paper explores a bio-inspired swarm confrontation algorithm specifically designed for complex hilly terrains in the context of electronic games. The novelty of the proposed algorithm lies in its utilization of biologically inspired strategies to facilitate adaptive and efficient decision-making in dynamic environments. Drawing from the collective hunting behaviors of various animal species, this paper distills two key confrontation strategies: focused fire for target selection and flanking encirclement for movement coordination and attack execution. These strategies are embedded into a decentralized swarm decision-making framework, enabling agents to exhibit enhanced responsiveness and coordination in complex gaming landscapes. To validate its effectiveness, extensive experiments were conducted, comparing the proposed approach against three established algorithms. The results demonstrate that this method achieves a confrontation win rate exceeding 80%, outperforming existing techniques in both engagement efficiency and survivability. Additionally, two novel performance indices, namely the average agent quantity loss rate and the average health loss rate, are introduced to provide a more comprehensive assessment of algorithmic effectiveness. Furthermore, the impact of key algorithmic parameters on performance indices is analyzed, offering insights into the adaptability and robustness of the proposed algorithm.

## 1. Introduction

With the advancement of technology, there is a growing demand for applications involving unmanned swarm cooperation and confrontational scenarios, not only in real-world robotics but also in virtual environments such as electronic games. Swarm confrontation represents a novel tactical paradigm that leverages the coordinated behavior of multiple unmanned aerial vehicles (UAVs) [1,2,3,4,5]. These algorithms have also found widespread application in gaming simulations, such as in StarCraft II [6], where complex agent coordination and strategic planning play a critical role in gameplay.

To enhance task execution efficiency and the success rate of agent swarms in complex and dynamic confrontational environments, a series of simulation methodologies for swarm confrontation strategies has been developed [7,8]. Evolutionary algorithms, such as particle swarm optimization and differential evolution, play a pivotal role in this research. These algorithms iteratively refine candidate solutions by emulating biological evolutionary mechanisms, including selection, crossover, and mutation, to converge toward optimal solutions. Multi-agent reinforcement learning (MARL), a subfield of reinforcement learning [9], focuses on developing strategies in environments where multiple agents coexist and interact. Each agent learns to perform optimal actions through collaboration or confrontation with other agents to achieve its objectives.

In recent years, swarm intelligence algorithms have seen substantial advancements [10] and have played a pivotal role in swarm confrontation. Reference [11] presented the mayfly algorithm, an optimization method inspired by mayflies’ behavior, combining swarm intelligence and evolutionary principles. Reference [12] presented a mathematical model that captures red fox behaviors such as foraging, hunting, population dynamics, and evading predators. By combining local and global optimization strategies with a reproduction mechanism, this model forms the basis of the red fox optimization algorithm. Reference [13] introduced the Flying Foxes Optimization (FFO) algorithm, drawing inspiration from the adaptive survival strategies of flying foxes in heatwaves. By incorporating fuzzy logic for dynamic parameter adjustment, FFO functions as a self-adaptive, parameter-free optimization technique. Reference [14] offered an innovative approach in swarm robotics, drawing inspiration from the foraging behavior of fish schools. By employing a bio-inspired neural network and a self-organizing map, the swarm replicates fish-like behaviors, including collision-free navigation and dynamic subgroup formation. Reference [15] explored the critical role of UAV swarms in the modern world, highlighting the urgent need for attack–defense-capable swarms. It introduced a bio-inspired decision-making method for UAV swarm confrontations using MARL, addressing the challenges of exponential training time as swarm size increases, drawing inspiration from natural group hunting behaviors.

This paper presents a bio-inspired confrontation algorithm aimed at improving success rates in swarm-based confrontations, particularly within the context of electronic games. Specifically, in a hilly environment, the undulating terrain obstructs the agents’ field of view, preventing them from fully acquiring real-time information about the opponent. Inspired by the hunting behaviors of various animal groups, such as lion prides and wild dog packs, two confrontation strategies are explored: the focused-fire strategy and flanking encirclement strategy. These strategies are integrated within hilly environments to develop a novel biomimetic swarm confrontation algorithm.

The contributions of this work are as follows:

- In contrast to purely 2D or 3D confrontation environments [15,16,17,18,19,20,21], this is the first time that a semi-3D confrontation environment, i.e., hilly terrain, has been considered regarding the swarm confrontation problem, which brings many challenges. First, the ability of the agent to gather information about opponents is limited. Second, virtual projectiles or actions executed by agents may be blocked by the terrain. Furthermore, the terrain constrains the agents’ postures, adding even more complexity to decision-making.
- Compared to agents that employ a particle model for movement [8,16,22,23,24], to suit the semi-3D confrontation environment, this paper adopts the unicycle model as a kinematic model of agents, which is more realistic yet complicated for confrontation scenarios. In addition, the rotating module responsible for targeting can freely spin on its supporting plane, while the elevation unit is capable of vertical adjustment. As a result, incorporating the additional degrees of freedom introduced by these rotational components leads to a more complex kinematic model compared to the standard unicycle model.
- Drawing on the behavioral characteristics exhibited by prides of lions and packs of wild dogs during their hunts, this paper proposes key algorithms suited to swarm confrontations. Compared with algorithms based on reinforcement learning or target-based assignment [15,25,26], the proposed approach focuses on specific behaviors throughout the confrontation, enhancing its interpretability and practical applicability—particularly in simulation-based environments such as electronic games. In direct comparisons against the aforementioned algorithms, the proposed method achieves a win rate exceeding 80%.
- For the evaluation of confrontation algorithms, in addition to traditional win rate assessment [24,25,27,28,29], two more performance indices are adopted, i.e., the agents’ quantity loss rate and the agents’ health loss rate. These two indices reflect the cost paid by the swarm confrontation algorithm to win from different perspectives, and the test results further highlight the superiority of the proposed bio-inspired swarm confrontation algorithm.

## 2. Related Work

### 2.1. Optimization Algorithms

In terms of evolutionary algorithms, reference [16] proposed an evolutionary algorithm (EA)-based attack strategy for swarm robots in denied environments, eliminating reliance on global positioning and communication. Each robot optimizes its movement using local sensing, evaluating threats and benefits through an EA-driven fitness function. With integrated collision avoidance, the swarm achieves effective collaboration and confrontation. Reference [30] introduced an evolutionary task allocation method for optimizing drone task distribution based on collaborative behavior, alongside a collaborative control method for UAVs to maintain formation during task execution. Reference [31] developed an optimized multi-UAV cooperative path planning approach for complex confrontation scenarios. A realistic threat model was developed, incorporating threat levels and fuel consumption constraints within a multi-objective optimization framework. Reference [32] proposed an evolutionary expert system tree for managing unexpected situations in aerial combat, while reference [33] introduced an enhanced particle swarm optimization algorithm that improves global search capabilities without adding computational complexity. Reference [34] examined strategic choices in a game-theoretic model of UAVs using a strategy evolution game, and reference [35] proposed an evolutionary optimization algorithm addressing the limitations of particle swarm optimization. Reference [36] expanded torch, a heterogeneous–homogeneous swarm coevolution method designed to enhance the evolutionary capabilities of swarm robots. Addressing the challenges of balancing evolutionary efficiency and strategy performance, torch employs a swarm coevolution mechanism to accelerate adaptation. A behavior expression tree is incorporated to expand the strategy search space, enabling more flexible and effective evolution. Reference [37] presented an improved differential evolution method based on Pareto optimal matching for multi-objective binary optimization problems. However, further optimization is required for complex environments with obstacles and multi-region challenges, and for integrating task allocation and collaborative control.

### 2.2. Multi-Agent Reinforcement Learning

MARL has seen significant advancements in recent years  [38,39]. Reference [40] proposed the hierarchical attention actor–critic (HAAC) algorithm to enhance decision-making in large-scale UAV swarm confrontations. By integrating a hierarchical actor policy with a centralized critic network based on the hierarchical two-stage attention network, HAAC captures UAV interactions and optimizes coordination. It effectively reduces state and action space complexity, improving scalability and outperforming existing methods in large-scale scenarios. Reference [41] proposed a one-vs-one within-visual-range air combat strategy generation algorithm based on a multi-agent deep deterministic policy gradient (MADDPG). The combat scenario is modeled as a two-player zero-sum Markov game, incorporating a target position prediction method to enhance decision-making. To bypass the constraints of basic fighter maneuvers, a continuous action space is adopted. Additionally, a potential-based reward shaping method improves learning efficiency. Reference [42] introduced a learning-based interception strategy for UAV territorial defense against invaders from various directions and speeds. The initial state’s impact on interception success was analyzed to define viable defense boundaries. Given the continuous action and state spaces, conventional decision methods face dimensionality issues. To address this, a fuzzy logic-enhanced actor–critic algorithm was proposed, effectively reducing computational complexity. To manage group situational complexity, reference [43] proposed a multi-agent transformer integrated with a virtual object network. Furthermore, reference [44] established two non-cooperative game models within the multi-agent deep reinforcement learning paradigm, successfully achieving Nash equilibrium in a five-on-five drone confrontation scenario. Reference [45] validated task allocation and decision-making in a simulation environment with mobile threats and targets. Reference [28] proposed a MARL approach that integrates macro actions and human expertise for UAV swarm decision-making. By modeling the swarm as a multi-agent system and using macro actions to address sparse rewards and large state-action spaces, the method enhances learning efficiency. Human-designed actions further optimize policies, enabling superior performance in complex confrontation scenarios. Lastly, reference [46] explored pursuit–evasion using deep reinforcement learning, where multiple homogeneous agents pursue an omnidirectional target under unicycle kinematics. A shared experience approach trains a policy for a fixed number of pursuers, executed independently at runtime.

Compared to the aforementioned algorithms, the proposed algorithm seamlessly integrates behaviors observed in animal confrontations into the confrontation process. It eliminates the need for model training and complex iterative computations while still delivering high performance.

Notation: R denotes the set of real numbers. For two vectors, *a* and *b*, a·b and a×b denote the inner product and cross product of *a* and *b*, respectively. For a nonzero vector *a*, M(a) is defined by

(1) $$M\left(a\right)=\frac{a}{∥a∥}.$$

For θ∈R, define the following rotation matrix R(θ):

(2) $$R\left(\theta \right)=\left[\begin{array}{ccc} \cos\theta & -\sin\theta & 0\\ \sin\theta & \cos\theta & 0\\ 0 & 0 & 1 \end{array}\right].$$

## 3. Problem Description

In this paper, we consider the swarm confrontation problem of two swarms of agents in hilly terrain. In particular, the two swarms of agents have equal quantities and abilities. This setting is especially relevant to electronic game simulations, where agents frequently engage in symmetric confrontational tasks within terrain-rich environments. In this section, descriptions of the hilly terrain and the agent model are given first, which are then followed by a description of the swarm confrontation problem.

### 3.1. Confrontation Environment

**Figure 1:**
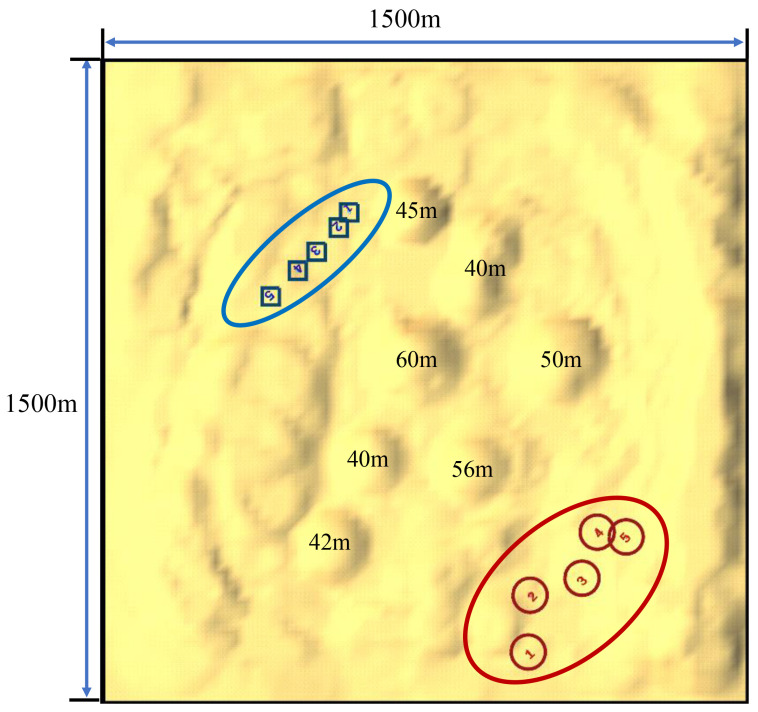
A panoramic view of the hilly terrain. As illustrated in the figure, red and blue agents labeled 1 to 5 are respectively positioned at the opposite corners of the terrain. This terrain comprises multiple rolling hills, with elevations ranging from 40 m to 60 m, and spans an area of 1500 m by 1500 m. The undulating topography includes lower-lying valleys and prominent hilltops.

A representative example of the hilly terrain used in this study for electronic game simulations is illustrated in Figure 1. Let $L_{1}$ and $L_{2}$ denote the length and width of the map, respectively, and let H represent the maximum height of the terrain. Note that agents can only move along the surface of the hilly terrain, which brings three challenges that have never been faced before. First, the ability of the agent to gather information about opponents is limited, as the hills may block the agent’s field of view, as shown by Figure 1. Second, the shells fired by the agents may be blocked by the terrain. Third, the terrain constrains the agents’ posture, making it difficult to aim.

### 3.2. Agent Model

In this paper, agents are categorized into red and blue teams. Suppose each team consists of *N* agents. For i=1,…,N, $r_{i}$ represents the *i*th agent on the red team, while $b_{i}$ represents the *i*th agent on the blue team. By default, the red team is equipped with the bio-inspired swarm confrontation algorithm, while the blue team is equipped with other existing swarm confrontation algorithms.

#### 3.2.1. Kinematics

**Figure 2:**
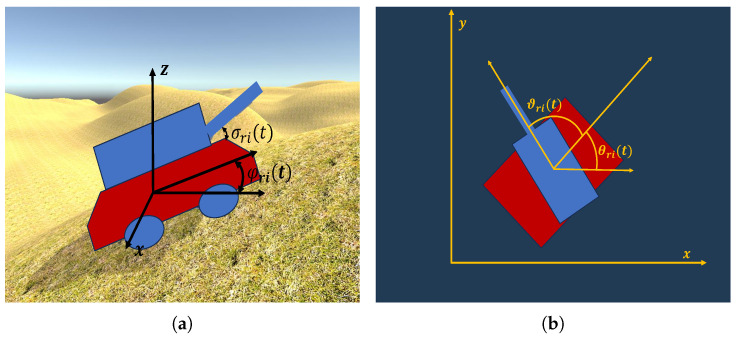
The angles of the agent. (**a**) The schematic illustration of the angles $\sigma_{ri}\left(t\right)$ and $\varphi_{ri}\left(t\right)$. (**b**) The schematic illustration of the angles $\theta_{ri}\left(t\right)$ and $\vartheta_{ri}\left(t\right)$.

The kinematic equations for agent $r_{i}$ are given by

(3) $$\begin{array}{cc} x_{ri}\left(t+1\right) & =x_{ri}\left(t\right)+v\cos\left(\theta_{ri}\left(t\right)\right)\sin\left(\varphi_{ri}\left(t\right)\right)\Delta t\\ y_{ri}\left(t+1\right) & =y_{ri}\left(t\right)+v\sin\left(\theta_{ri}\left(t\right)\right)\sin\left(\varphi_{ri}\left(t\right)\right)\Delta t\\ z_{ri}\left(t+1\right) & =z_{ri}\left(t\right)+v\cos\left(\varphi_{ri}\left(t\right)\right)\Delta t\\ \theta_{ri}\left(t+1\right) & =\theta_{ri}\left(t\right)+\omega_{ri}\left(t\right)\Delta t\\ \vartheta_{ri}\left(t+1\right) & =\vartheta_{ri}\left(t\right)+\Omega_{ri}\left(t\right)\Delta t\\ \sigma_{ri}\left(t+1\right) & =\sigma_{ri}\left(t\right)+\Phi_{ri}\left(t\right)\Delta t \end{array}$$

where $p_{ri}\left(t\right)={\left(x_{ri}\left(t\right),y_{ri}\left(t\right),z_{ri}\left(t\right)\right)}^{T}\in R^{3}$ represents the position of agent $r_{i}$ at time *t*; v∈R represents the linear speed; $\theta_{ri}\left(t\right)\in R$ and $\omega_{ri}\left(t\right)\in R$ represent the heading angle and angular velocity of the body, respectively; $\varphi_{ri}\left(t\right)\in R$ represents the pitch angle of the body, which is determined by the topography; $\vartheta_{ri}\left(t\right)\in R$ and $\Omega_{ri}\left(t\right)\in R$ denote the heading angle and angular velocity of the rotating module, respectively, while $\sigma_{ri}\left(t\right)\in R$ and $\Phi_{ri}\left(t\right)\in R$ represent the pitch angle and angular velocity of the elevation unit, respectively; and Δt denotes the sampling time. The various aforementioned angles are illustrated by Figure 2. Additionally, $\omega_{m}$, $\Omega_{m}$, and $\Phi_{m}$ denote the maximum rotational speed for $\omega_{ri}\left(t\right)$, $\Omega_{ri}\left(t\right)$, and $\Phi_{ri}\left(t\right)$, respectively. In this paper, $\omega_{ri}\left(t\right),\Omega_{ri}\left(t\right),\Phi_{ri}\left(t\right)$ are considered to be the control inputs of agent $r_{i}$. These control inputs will be specified later by the proposed bio-inspired swarm confrontation algorithm. At the onset of the confrontation, the initial parameters are configured as follows: $x_{ri}\left(0\right)\in \left[0,L_{1}\right],y_{ri}\left(0\right)\in \left[0,L_{2}\right],z_{ri}\left(0\right)\in \left[0,H\right],\theta_{ri}\left(0\right)=0^{\circ }$, $\varphi_{ri}\left(0\right)=0^{\circ }$, $\vartheta_{ri}\left(0\right)=0^{\circ }$, $\sigma_{ri}\left(0\right)=0^{\circ }$. Similarly, we can define $p_{bi}\left(t\right),\theta_{bi}\left(t\right),\omega_{bi}\left(t\right),\vartheta_{bi}\left(t\right),\Omega_{bi}\left(t\right),\sigma_{bi}\left(t\right),\Phi_{bi}\left(t\right)$ for agent $b_{i}$, and the details are omitted. Note that the agents of the blue team have the same linear speed *v* and maximal rotational speeds ( $\omega_{m}$, $\Omega_{m}$, and $\Phi_{m}$) as the agents of the red team.

#### 3.2.2. Information Acquisition

**Figure 3:**
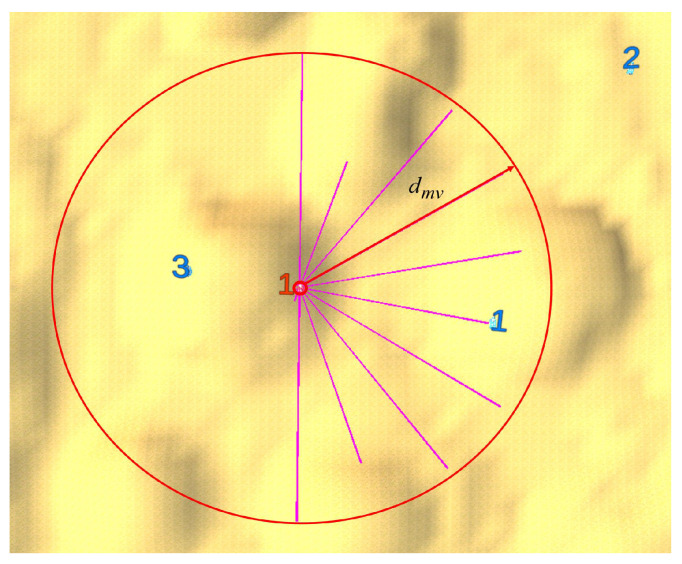
Agent $r_{1}$ uniformly emits rays for detection. As illustrated in the figure, red agent $r_{1}$ emits rays toward blue agents $b_{1}$, $b_{2}$, and $b_{3}$ for detection. These rays successfully detect $b_{1}$ but fail to reach $b_{2}$ and $b_{3}$. Specifically, $b_{2}$ is beyond the maximum detection range, while $b_{3}$ is obstructed by the terrain. Therefore, as defined by Equation (4), $b_{1}$ is a member of the set $N_{ri}\left(t\right)$, whereas $b_{2}$ and $b_{3}$ are not included.

During confrontation, an agent detects opponents by uniformly emitting rays, as shown by Figure 3. Define $d_{b_{j}}^{r_{i}}\left(t\right)=∥p_{ri}\left(t\right)-p_{bj}\left(t\right)∥$. The set of opponents whose information can be obtained by agent $r_{i}$ at time *t* is defined as follows:

(4) $$N_{ri}\left(t\right)=\left\{b_{j}|d_{b_{j}}^{r_{i}}\left(t\right)\leq d_{mv}\;\mathrm{and}\;\mathrm{the}\;\mathrm{ray}\;\mathrm{from}\;r_{i}\;\mathrm{to}\;b_{j}\;\mathrm{is}\;\mathrm{not}\;\mathrm{obstructed}\;\mathrm{by}\;\mathrm{the}\;\mathrm{terrain}\right\}$$

where $d_{mv}$ denotes the maximum detection range of the ray. Similarly, $N_{bi}\left(t\right)$ denotes the set of opponents whose information is accessible to agent $b_{i}$ at time *t*.

Note that on the one hand, the rays can only detect within the maximum detection range $d_{mv}$, and on the other hand, the rays can be obstructed by hills. For agent $r_{i}$, it can acquire the following information at time *t*.

- The positions of all the surviving agents of the red team at time *t*.
- The positions of all the surviving agents of the blue team belonging to the set $N_{ri}\left(t\right)$.

The method of information acquisition for the agents of the blue team is the same.

#### 3.2.3. Attack and Damage

Agents engage opponents by launching projectiles with an initial speed of $v_{p}$, which subsequently follow a ballistic trajectory under the influence of gravity. The direction of the projectile is determined by the posture of the elevation unit. Each agent starts with an initial health point (HP), and once hit by a shell, the health point is reduced by $h_{d}$. Let $h_{ri}\left(t\right)$ denote the health point of $r_{i}$ at time *t*. If $h_{ri}\left(t\right)\leq 0$, agent $r_{i}$ is considered destroyed. Moreover, the minimum firing interval between two attacks is $t_{cm}$, and the swarm confrontation algorithm will decide when to fire. Similarly, we can define $h_{bi}\left(t\right)$ for $b_{i}$.

### 3.3. Winning of the Confrontation

At the beginning of the confrontation, the red and blue teams are positioned at opposite corners of the map. Winning is declared for the side that annihilates all the agents of the opposing side within the time limit $t_{m}$. If all the agents are destroyed within $t_{m}$, or neither team wins within $t_{m}$, it is called a tie.

### 3.4. Algorithm Performance Indices

To assess the performance of the algorithm, three algorithm performance indices are considered in this paper, namely, the win rate, average agent quantity loss rate, and average agent health loss rate, which are detailed as follows. Consider a series of *M* matches between the red and blue teams. For the red team, let $M_{w}^{r}$ denote the number of matches won by the red team, and $H_{s}^{r}$ represent the initial health points of all members of the red team. For $k=1,\ldots ,M_{w}^{r}$, define $n_{k}^{r}$ and $h_{sk}^{r}$ as the number of agents lost by the red team and the total health points lost by the red team in the *k*th winning match, respectively. Then, the performance indices for the red team’s algorithm are established as follows:

- Winning rate $W_{r}$:
  (5) $$W_{r}=\frac{M_{w}^{r}}{M}$$
- Average agent quantity loss rate $\xi_{r}$:
  (6) $$\xi_{r}=\frac{1}{M_{w}^{r}}\sum_{k=1}^{M_{w}^{r}}\frac{n_{k}^{r}}{N}$$
- Average agent health loss rate $\lambda_{r}$:
  (7) $$\lambda_{r}=\frac{1}{M_{w}^{r}}\sum_{k=1}^{M_{w}^{r}}\frac{h_{sk}^{r}}{H_{s}^{r}}$$

The parameters $W_{b}$, $\xi_{b}$, and $\lambda_{b}$ can be similarly defined for the blue team following the same approach.

## 4. Bio-Inspired Swarm Confrontation Algorithm Design

Based on biologically inspired algorithms, agents must primarily address two key issues during the swarm confrontation process: selecting attack targets and making decisions regarding movement during the confrontation. This chapter begins by analyzing animal group behavior, summarizing the corresponding confrontation algorithms, and then connecting these algorithms to real confrontation scenarios for implementation.

### 4.1. Bio-Inspired Rules

**Figure 4:**
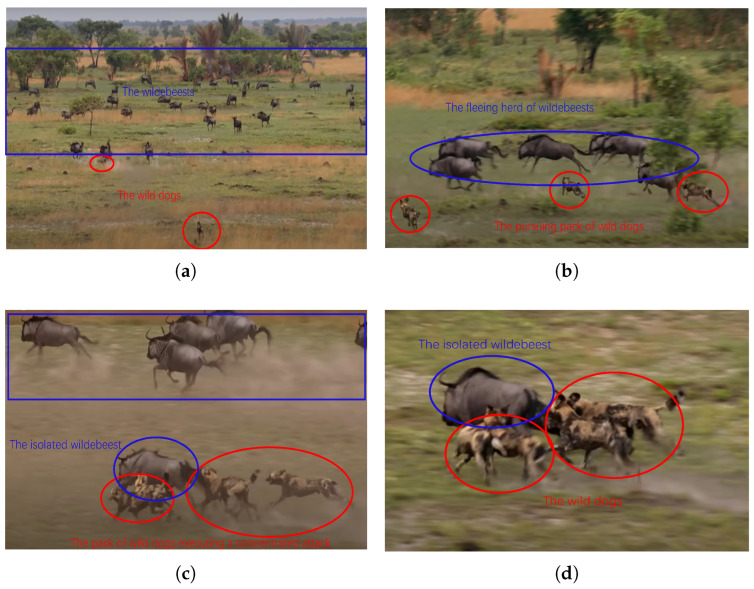
The focused-fire strategy is illustrated by the confrontation between a pack of wild dogs and a group of wildebeests [47]. (**a**) The wild dogs spot the wildebeest group and quickly close in, attempting to scatter them. (**b**) The wildebeests begin to flee, with the wild dogs in pursuit. (**c**) During the escape, a smaller, isolated individual emerges from the wildebeest group, becoming the target for the wild dogs. (**d**) The wild dogs then concentrate their efforts and launch an attack.

We employ the following analysis to address the problem of target selection during each agent’s confrontation process. As illustrated in Figure 4, a pack of wild dogs spots a group of wildebeests and swiftly closes in, attempting to scatter them. The wildebeests initially cluster together to confront the predators but soon become startled and begin to flee, with the wild dogs in pursuit. During the chase, a smaller, isolated individual emerges from the group, becoming the focus of the wild dogs’ attention. The pack then concentrates its efforts on launching an attack on the vulnerable wildebeest.

For the wild dogs, each individual is smaller in size and weaker in strength compared to a wildebeest. When the wildebeests cluster together, it becomes difficult for the wild dogs to inflict damage. Therefore, when an isolated individual appears within the wildebeest group, the wild dogs quickly shift their target, creating a situation where the many overpower the few, effectively completing the hunt. Drawing on the collective hunting behavior of a wild dog pack, agents in a hilly-terrain confrontation can switch attack targets based on the opponent’s position. If an opponent is far from its group, it becomes the priority target. This tactic results in a localized numerical advantage, allowing agents to eliminate the target quickly. We refer to this behavior as the focused-fire strategy.

**Figure 5:**
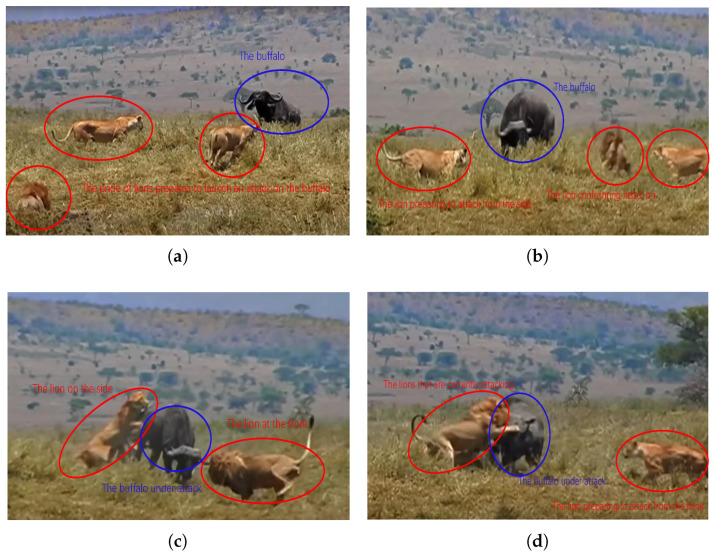
The flanking encirclement strategy is illustrated by the confrontation between a lion pride and a buffalo herd [48]. (**a**) Three lions seize the opportunity to attack a buffalo, with the pride approaching in a triangular formation. (**b**) The central lion confronts the buffalo head-on while the lions on both sides flank the buffalo to form a pincer. (**c**) The lions complete the encirclement and launch an attack. (**d**) The lions successfully complete the hunt.

Efficient confrontation algorithms must select targets judiciously and make real-time decisions during the confrontation, adjusting their movement direction based on the evolving situation. This section further analyzes the group attack behavior of animals. As shown in Figure 5, three lions seize the opportunity to attack a buffalo, approaching it in a triangular formation. The central lion confronts the buffalo head-on, while the lions on both sides maneuver to flank it, forming a pincer movement. After completing the encirclement, the lions launch their attack and complete the hunt.

If the lion pride were to attack head-on as a group, the buffalo, sensing danger, would likely counterattack or flee, which could result in casualties among the lions or allow the buffalo to escape. The pride increases its chances of a successful hunt by attacking from multiple directions. In an agent-based confrontation, if two or more agents target the same opponent, one agent can engage the opponent head-on while the others flank from the sides, efficiently neutralizing the target. We refer to this behavior as the flanking encirclement strategy.

### 4.2. Design of Swarm Confrontation Algorithm

After analyzing and adapting bio-inspired rules, these principles need to be applied to practical confrontation algorithms. The design of the confrontation algorithm is mainly divided into three parts: target selection, motion planning, and automatic aiming. Taking the red agent $r_{i}$ as an example, the following sections detail the design of these three components.

#### 4.2.1. Target Selection

Inspired by the hunting behavior of wild dogs in nature, the target selection algorithm employs the focused-fire strategy. Define $d_{r_{k}}^{r_{i}}\left(t\right)=∥p_{ri}\left(t\right)-p_{rk}\left(t\right)∥$. Let $n_{b_{a}}^{r_{i}}\left(t\right)$ represent the number of surviving opponents detectable by $r_{i}$, and let $p_{c_{b}}^{r_{i}}\left(t\right)$ denote the central position of these opponents. Let $I_{x}^{r_{i}}\left(t\right)$ denote the label of the *x*th closest surviving opponent to $r_{i}$, and let $T_{ri}\left(t\right)$ denote the label of the attack target chosen by $r_{i}$. Let $c_{t}$ be a positive integer and $d_{f}$ be a positive real number. The target selection algorithm is described by Algorithm 1.
**Algorithm 1** Target Selection Algorithm1:**input**: $n_{b_{a}}^{r_{i}}\left(t\right)$—the number of surviving opponents detectable by $r_{i}$,2:            $p_{c_{b}}^{r_{i}}\left(t\right)$—the central position of these detected opponents.3:**output**: $T_{ri}\left(t\right)$—the label of the attack target chosen by $r_{i}$.4:**set** x=15:**if** $n_{b_{a}}^{r_{i}}\left(t\right)=0$ **then**6:     $T_{ri}\left(t\right)=null$7:**else**8:     C=true9:     **set** $o_{j}^{r_{i}}=0,\;\forall j\in \left\{1,2,\ldots ,N\right\}$10:     **while** $x\leq n_{b_{a}}^{r_{i}}\left(t\right)$ **and** C **do**11:         $l=I_{x}^{r_{i}}\left(t\right)$12:         **if** $∥p_{c_{b}}^{r_{i}}\left(t\right)-p_{bl}\left(t\right)∥>d_{f}$ **then**13:            $T_{ri}\left(t\right)=l$14:            C=false15:         **else**16:            **set** c=0,k=117:            **while** k≤N **do**18:                **if** $r_{k}$ is surviving and k≠i **then**19:                   **if** $o_{k}^{r_{i}}=0$ **then**20:                   **if** $d_{bl}^{r_{i}}\left(t\right)>d_{bl}^{r_{k}}\left(t\right)$ **then**21:                          c=c+122:                          $o_{k}^{r_{i}}=1$23:            **if** $c>c_{t}$ **then**24:               x=x+125:            **else**26:               $T_{ri}\left(t\right)=l$27:               C=false28:        **if** $x>n_{b_{a}}^{r_{i}}\left(t\right)$ **then**29:            $T_{ri}\left(t\right)=I_{1}^{r_{i}}\left(t\right)$

**Figure 6:**
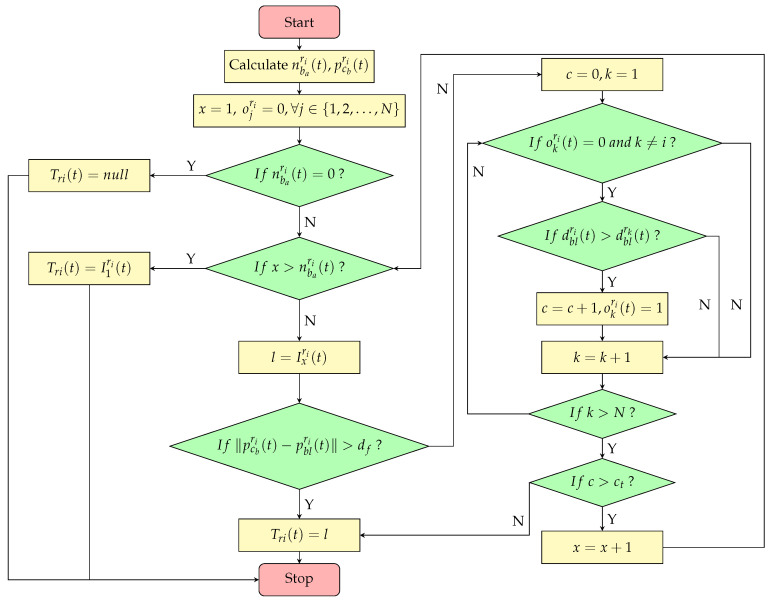
A flowchart of the target selection algorithm.

According to Algorithm 1, $n_{b_{a}}^{r_{i}}\left(t\right)$ and $p_{c_{b}}^{r_{i}}\left(t\right)$ serve as input parameters, while $T_{ri}\left(t\right)$ functions as the output parameter. The target selection algorithm follows a multi-level decision-making process. First, after obtaining $I_{x}^{r_{i}}\left(t\right)$, $r_{i}$ evaluates the spatial distribution of its opponents. If the distance between $b_{l}$ and the center of visible opponents within $r_{i}$’s range exceeds $d_{f}$, $b_{l}$ is considered to have deviated from its team formation, and $r_{i}$ prioritizes attacking $b_{l}$. Second, as indicated in steps 10 to 27 of Algorithm 1, these steps involve an iterative computation process, with $c_{t}$ playing a crucial role in the iteration. If $b_{l}$ is positioned closer to its own team, $r_{i}$ determines its relative ranking within the team based on proximity to $b_{l}$. If $r_{i}$ ranks beyond $c_{t}$, it must recalculate $I_{x}^{r_{i}}\left(t\right)$ and repeat this process iteratively until its ranking falls within $c_{t}$. This design helps prevent the excessive concentration of attack targets among red agents, thereby reducing resource overflow and minimizing wastage. Finally, if no opponent within $r_{i}$’s field of view meets the above conditions, $r_{i}$ selects the nearest opponent as its attack target, denoted as $I_{1}^{r_{i}}\left(t\right)$. As described above, the algorithm not only prevents an excessive number of agents from attacking the same target, thereby avoiding unnecessary concentration of launched projectiles, but also creates a local numerical advantage. This demonstrates the focused-fire strategy proposed in this paper, and a flowchart of the algorithm is shown in Figure 6.

#### 4.2.2. Motion Planning

Incorporating the competitive behavior of biological swarms into an agent’s confrontation process primarily involves planning its trajectory. Given that the field is undulating and there are no complex obstacles, we implement the agent’s path planning using the artificial potential field method. Considering that the agent also needs to avoid obstacles presented by teammates in the environment, the agent’s direction of movement can be decomposed into the sum of two vectors.

(1) Consider the motion planning of $r_{i}$ in the absence of obstacles. When $T_{ri}\left(t\right)=\mathrm{null}$, $r_{i}$ selects the nearest hilltop, denoted as $p_{m}^{ri}\left(t\right)$, as its movement target to facilitate opponent searching. Conversely, when $T_{ri}\left(t\right)\neq \mathrm{null}$, $r_{i}$ selects $p_{b_{T}}^{r_{i}}\left(t\right)$ as its movement target. Here, $p_{b_{T}}^{r_{i}}\left(t\right)$ denotes the position of the opponent labeled $T_{ri}$, which has been assigned according to Algorithm 1. The movement direction toward the target is defined as follows:

(8) $$G_{o}^{ri}\left(t\right)=\left\{\begin{array}{cc} p_{m}^{r_{i}}\left(t\right)-p_{r_{i}}\left(t\right) & T_{ri}\left(t\right)=null\\ p_{b_{T}}^{r_{i}}\left(t\right)-p_{r_{i}}\left(t\right) & T_{ri}\left(t\right)\neq null \end{array}\right.$$

In a hunt, a pride of lions typically attacks prey from multiple directions. The lions at the front often feint to distract the prey while the lions on the flanks wait for an opportunity to strike. Inspired by this behavior, agents can employ a flanking encirclement strategy during confrontations by setting different movement directions.

The following section introduces the method for determining the relative position of $r_{i}$ within the team. Let $\rho_{r_{i}}\left(t\right)$ represents the relative position of $r_{i}$ within the friendly team that shares the same opponent. When $\rho_{r_{i}}\left(t\right)=0$, $r_{i}$ is in the middle; when $\rho_{r_{i}}\left(t\right)=1$, $r_{i}$ is on the left side; and when $\rho_{r_{i}}\left(t\right)=-1$, $r_{i}$ is on the right side. The method for obtaining $\rho_{r_{i}}\left(t\right)$ is presented as follows:

(9) $$l_{ri}=M\left(p_{b_{T}}^{r_{i}}\left(t\right)-p_{c}^{r_{i}}\left(t\right)\right)$$

(10) $$d_{ri}\left(t\right)=\left(p_{r_{i}}\left(t\right)-p_{c}^{r_{i}}\left(t\right)\right)\;\cdot \;\frac{l_{ri}\times l_{z}}{∥l_{ri}\times l_{z}∥}$$

(11) $$\rho_{r_{i}}\left(t\right)=\left\{\begin{array}{cc} -1 & \;d_{ri}\left(t\right)>\epsilon_{1},\\ 0 & \;-\epsilon_{1}\leq d_{ri}\left(t\right)\leq \epsilon_{1},\\ 1 & \;d_{ri}\left(t\right)<-\epsilon_{1}. \end{array}\right.$$

where $p_{c}^{r_{i}}\left(t\right)$ represents the position of the agent closest to $p_{b_{T}}^{r_{i}}\left(t\right)$ among the group of agents sharing the same attack target. Meanwhile, $d_{ri}\left(t\right)$ denotes the projected offset of $r_{i}$ within the team, and $\epsilon_{1}$ is the reference value used to determine the position interval. $l_{z}$ denotes the unit direction vector along the *z*-axis. The actual movement direction $G_{ri}\left(t\right)$ of $r_{i}$ in an obstacle-free environment is obtained by multiplying $\rho_{r_{i}}\left(t\right)$ by the rotation angle $\theta_{s}$ and applying the resulting rotation matrix to $G_{o}^{ri}\left(t\right)$. In the case where $T_{ri}\left(t\right)=\mathrm{null}$, $G_{ri}\left(t\right)$ is directly equivalent to $G_{o}^{ri}\left(t\right)$.

(2) Calculate the vector $X_{k}^{r_{i}}\left(t\right)$ between teammates $p_{r_{k}}\left(t\right)$ and $p_{r_{i}}\left(t\right)$ within the obstacle avoidance range $d_{a}$. Since closer teammates require stronger obstacle avoidance force, the resulting vector should be larger. Therefore, it is necessary to normalize the vector and apply weighting. This algorithm selects $1/d_{r_{k}}^{r_{i}}\left(t\right)$ as the weight for each vector, and finally, the sum of all vectors, denoted as $X_{ri}\left(t\right)$, is obtained:

(12) $$X_{k}^{r_{i}}\left(t\right)=\left\{\begin{array}{cc} \frac{p_{r_{i}}\left(t\right)-p_{r_{k}}\left(t\right)}{{\left(d_{r_{k}}^{r_{i}}\left(t\right)\right)}^{2}} & \mathrm{if}\;0<d_{r_{k}}^{r_{i}}\left(t\right)\leq d_{a}\;\mathrm{and}\;r_{k}\;\mathrm{is}\;\mathrm{alive},\\ 0 & \mathrm{otherwise}. \end{array}\right.$$

(13) $$X_{ri}\left(t\right)=\sum_{k=1}^{N}X_{k}^{r_{i}}\left(t\right)$$

Since the influence of each vector on the agent’s movement is different, each vector needs to be normalized and weighted to obtain the final direction of movement $F_{ri}\left(t\right)$:

(14) $$F_{ri}\left(t\right)=k_{1}M\left(G_{ri}\left(t\right)\right)+k_{2}M\left(X_{ri}\left(t\right)\right)$$

where $k_{1}$ and $k_{2}$ denote the weight coefficients assigned to each vector.

Let $t_{c}^{r_{i}}$ represent the time elapsed since $r_{i}$ fired its last shell. $d_{bc_{1}}$ represents the maximum distance threshold for $r_{i}$ to execute a retreating flanking encirclement strategy, while $d_{bc_{2}}$ represents the minimum distance threshold for $r_{i}$ to execute a flanking maneuver during an advance, as well as the minimum retreat distance for flanking when $t_{c}^{r_{i}}<t_{cm}$. $d_{a}$ represents the distance for avoiding teammates. $\theta_{F}^{r_{i}}\left(t\right)$ represents the heading angle of $F_{ri}\left(t\right)$. $e_{\theta 1}^{r_{i}}\left(t\right)$ and $e_{\theta 2}^{r_{i}}\left(t\right)$ denote the deviation angles between the current movement direction and the final target direction in the clockwise and counterclockwise directions, respectively. The detailed implementation is presented in Algorithm 2.
**Algorithm 2** Motion Planning Algorithm1:**input** $G_{o}^{ri}\left(t\right)$2:**output** $\omega_{ri}\left(t\right)$3:**if** $T_{ri}\left(t\right)=null$ **then**4:     $G_{ri}\left(t\right)=G_{o}^{ri}\left(t\right)$5:**else**6:     calculate the $\rho_{r_{i}}\left(t\right)$ by Equations (9)–(11)7:     **if** $\left(d_{b_{T}}^{r_{i}}\left(t\right)<d_{bc_{1}}\right)\;\mathrm{or}\;\left(d_{bc_{1}}<d_{b_{T}}^{r_{i}}\left(t\right)\leq d_{bc_{2}}\;\mathrm{and}\;t_{c}^{r_{i}}<t_{cm}\right)$ **then**8:        $G_{ri}\left(t\right)=-R\left(-\theta_{s}\;\rho_{r_{i}}\left(t\right)\right)G_{o}^{ri}$9:     **else**10:        $G_{ri}\left(t\right)=R\left(\theta_{s}\;\rho_{r_{i}}\left(t\right)\right)G_{o}^{ri}$11:calculate the $X_{ri}\left(t\right)$ by Equations (12) and (13)12:calculate the $F_{ri}\left(t\right)$ by Equation (14)13:$e_{\theta 1}^{r_{i}}\left(t\right)=\left(\theta_{ri}\left(t\right)-\theta_{F}^{r_{i}}\left(t\right)\right)\;\mathrm{mod}\;\left(2\pi \right),\;e_{\theta 2}^{r_{i}}\left(t\right)=\left(\theta_{F}^{r_{i}}\left(t\right)-\theta_{ri}\left(t\right)\right)\;\mathrm{mod}\;\left(2\pi \right)$14:$\omega_{ri}\left(t\right)=\mathrm{sign}\left(e_{\theta 2}^{r_{i}}\left(t\right)-e_{\theta 1}^{r_{i}}\left(t\right)\right)\omega_{m}$

**Figure 7:**
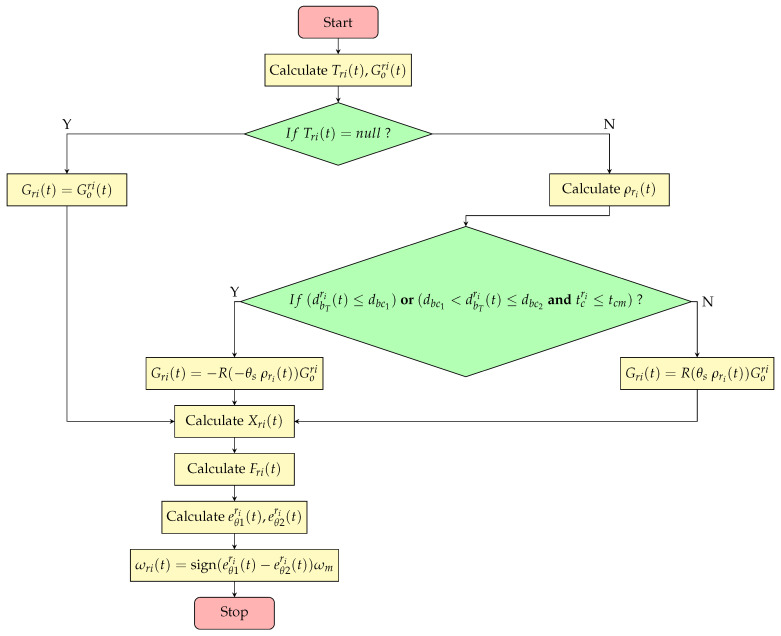
A flowchart of the motion planning algorithm.

According to Algorithm 2, when $r_{i}$ detects opponents, it first computes $G_{o}^{ri}\left(t\right)$ and then determines its relative position $\rho_{r_{i}}\left(t\right)$ among teammates that share the same attack target. Based on $\rho_{r_{i}}\left(t\right)$, $r_{i}$ adjusts the direction of $G_{o}^{ri}\left(t\right)$. If $r_{i}$ is positioned on the right side of the formation, $G_{o}^{ri}\left(t\right)$ is rotated clockwise by $\theta_{s}$ degrees; if it is on the left side, the rotation is counterclockwise by $\theta_{s}$ degrees. If $r_{i}$ is centrally positioned within the formation, its movement direction remains unchanged. In scenarios where only two red agents share the same attack target, it suffices to determine the relative position of the agent positioned farther from the target and assign it the appropriate movement direction. When $r_{i}$ is within a distance of $d_{bc_{1}}$ from the attack target, or if its firing cooldown is active while being within $d_{bc_{2}}$, its movement direction is set to retreat. Based on the previous steps, agents can be assigned to either direct confrontation or flanking maneuvers, enabling them to attack opponents from multiple angles. This approach is referred to as the flanking encirclement strategy. The critical steps of this strategy are outlined in steps 6 to 10 of Algorithm 2. As a result, $G_{ri}\left(t\right)$ is determined. Then, incorporating the obstacle avoidance vector $X_{ri}\left(t\right)$ yields the final movement direction $F_{ri}\left(t\right)$. The corresponding flowchart of the algorithm is shown in Figure 7.

#### 4.2.3. Automatic Aiming Algorithm

In the following, taking $r_{i}$ as an example, the motion process of the rotating module and the elevation unit after determining the attack target $T_{ri}\left(t\right)$ is introduced. Upon identifying $T_{ri}\left(t\right)$, $r_{i}$ adjusts $\vartheta_{ri}\left(t\right)$ and $\sigma_{ri}\left(t\right)$, based on the relative angle between the target and its position, thereby achieving target aiming. When $r_{i}$ calculates the vector $u_{o}^{r_{i}}\left(t\right)$ from itself to the opponent, it then computes the angle $\theta_{tur}^{r_{i}}\left(t\right)$ between $u_{o}^{r_{i}}\left(t\right)$ and the rotating module’s direction vector $u_{tur}^{r_{i}}\left(t\right)$ in the XOY plane, rotating the rotating module left or right to make $\theta_{tur}^{r_{i}}\left(t\right)$ approach 0. Additionally, $r_{i}$ determines the angle $\theta_{bar}^{r_{i}}\left(t\right)$ between $u_{o}^{r_{i}}\left(t\right)$ and the unit direction vector of the elevation unit $u_{bar}^{r_{i}}\left(t\right)$, simultaneously rotating the elevation unit up or down to make $\theta_{bar}^{r_{i}}\left(t\right)$ approach 0. $\epsilon_{2}$ denotes the deviation range between the target angle and the actual angle. $f_{a}^{r_{i}}\left(t\right)$ serves as a flag indicating whether $r_{i}$ is actively aiming at an opponent. The specific implementation process is shown in Algorithm 3.
**Algorithm 3** Automatic Aiming Algorithm1:**input** $T_{ri}\left(t\right),u_{tur}^{r_{i}}\left(t\right),u_{bar}^{r_{i}}\left(t\right)$2:**output** $\Omega_{ri}\left(t\right),\Phi_{ri}\left(t\right),f_{a}^{r_{i}}\left(t\right)$3:**if** $T_{ri}\left(t\right)\neq null$ **then**4:     $u_{o}^{r_{i}}\left(t\right)=M\left(T_{ri}\left(t\right)-p_{r_{i}}\left(t\right)\right)$5:     $u_{o1}^{r_{i}}\left(t\right)=\left(u_{o_{x}}^{r_{i}},u_{o_{y}}^{r_{i}},0\right),u_{tur1}^{r_{i}}\left(t\right)=\left(u_{{tur}_{x}}^{r_{i}},u_{{tur}_{y}}^{r_{i}},0\right)$6:     $\theta_{tur}^{r_{i}}\left(t\right)=\arccos\left(\frac{u_{o1}^{r_{i}}\left(t\right)\;\cdot \;u_{tur1}^{r_{i}}\left(t\right)}{∥u_{o1}^{r_{i}}\left(t\right)∥\;∥u_{tur1}^{r_{i}}\left(t\right)∥}\right)\mathrm{sign}\left(l_{z}\;\cdot \;\left(\theta_{tur}^{r_{i}}\left(t\right)\times u_{o1}^{r_{i}}\left(t\right)\right)\right)$7:     $\Omega_{ri}\left(t\right)=\frac{\theta_{tur}^{r_{i}}\left(t\right)\Omega_{m}}{\pi }$8:     **if** $\theta_{tur}^{r_{i}}\left(t\right)<\epsilon_{2}$ **then**9:         $\theta_{bar}^{r_{i}}\left(t\right)=\arccos\left(\frac{u_{o}^{r_{i}}\left(t\right)\;\cdot \;u_{bar}^{r_{i}}\left(t\right)}{∥u_{o}^{r_{i}}\left(t\right)∥\;∥u_{bar}^{r_{i}}\left(t\right)∥})\right)\mathrm{sign}\left(u_{o_{y}}\left(t\right)-u_{bar_{y}}^{r_{i}}\left(t\right)\right)$10:        $\Phi_{ri}\left(t\right)=\frac{\theta_{bar}^{r_{i}}\left(t\right)\Phi_{m}}{\pi }$11:        **if** $\theta_{tur}^{r_{i}}\left(t\right)<\epsilon_{2}$ **and** $\theta_{bar}^{r_{i}}\left(t\right)<\epsilon_{2}$ **then**12:            $f_{a}^{r_{i}}\left(t\right)=1$13:        **else**14:            $f_{a}^{r_{i}}\left(t\right)=0$15:     **else**16:        $f_{a}^{r_{i}}\left(t\right)=0$

#### 4.2.4. Bio-Inspired Swarm Confrontation Algorithm

At the beginning of the confrontation, each agent determines its attack target using Algorithm 1. Then, it calculates its actual movement direction using Algorithm 2. Finally, Algorithm 3 is executed to precisely align with the target. During movement, the agent continuously assesses whether the conditions for firing are met and proceeds with the attack when appropriate. If all opponents are eliminated, the confrontation ends. Otherwise, Algorithms 1–3 are re-executed to recalculate the strategy.

**Figure 8:**
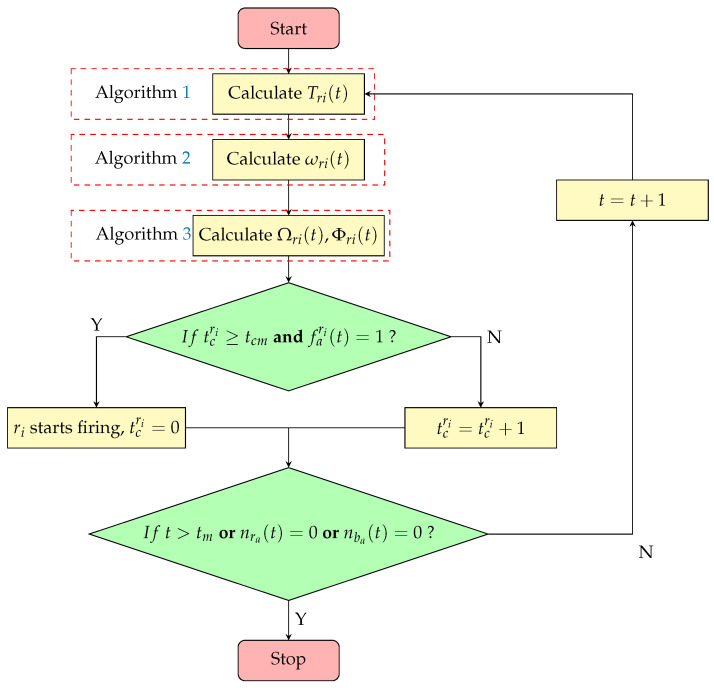
A flowchart of the bio-inspired swarm confrontation algorithm.

By integrating the algorithm designs discussed above, the final pseudo-code and a flowchart of the bio-inspired swarm confrontation algorithm are established, and are presented in Algorithm 4 and Figure 8. $n_{r_{a}}\left(t\right)$ and $n_{b_{a}}\left(t\right)$ represent the total number of surviving agents for the red and blue teams, respectively. Additionally, the entire process is carried out sequentially within time step *t*.
**Algorithm 4** Bio-inspired Swarm Confrontation Algorithm1:**for** step *t* **do**2:     execute Algorithm 13:     execute Algorithm 24:     execute Algorithm 35:     **if** $t_{c}^{r_{i}}>t_{cm}$ **and** $f_{a}^{r_{i}}\left(t\right)=1$ **then**6:        agent starts firing7:        $t_{c}^{r_{i}}=0$8:     **else**9:        $t_{c}^{r_{i}}=t_{c}^{r_{i}}+1$10:     **if** $t>t_{m}$ **or** $n_{r_{a}}\left(t\right)=0$ **or** $n_{b_{a}}\left(t\right)=0$ **then**11:        algorithm terminates12:     **else**13:        t=t+1

### 4.3. Algorithm Complexity Analysis

The bio-inspired confrontation algorithm presented in this paper consists primarily of three components: target selection, motion planning, and automatic aiming. The computational complexity of the automatic aiming algorithm is O(1), while the complexities of the other components are as follows:

(1) Target selection: Calculating the closest opponent to the agent has a complexity of O(N). Recalculating the opponent based on local principles has a complexity of O(mN), where *m* represents the number of recalculations required, m∈[1,N]. Calculating the centroid of opponents within the agent’s field of view has a complexity of O(N).

(2) Motion planning: Determining the agent’s position relative to the same opponent group has a complexity of O(N). Calculating the combined vector for teammate obstacle avoidance also has a complexity of O(N). Similarly, calculating the combined vector for opponent obstacle avoidance is O(N).

The overall algorithmic complexity is O(N) (best case) to $O(N^{2})$ (worst case).

## 5. Result Analysis

Comparative algorithms within the current environment must be introduced and adapted to evaluate the effectiveness of the swarm confrontation algorithm proposed in this paper. The comparative algorithms selected are the MARL Based on Biomimetic Action Space algorithm [15], the Consensus-Based Auction (CBA) algorithm [25], and the Assign Nearest (AN) algorithm [26].

### 5.1. Results Analysis for a Single Match

**Figure 9:**
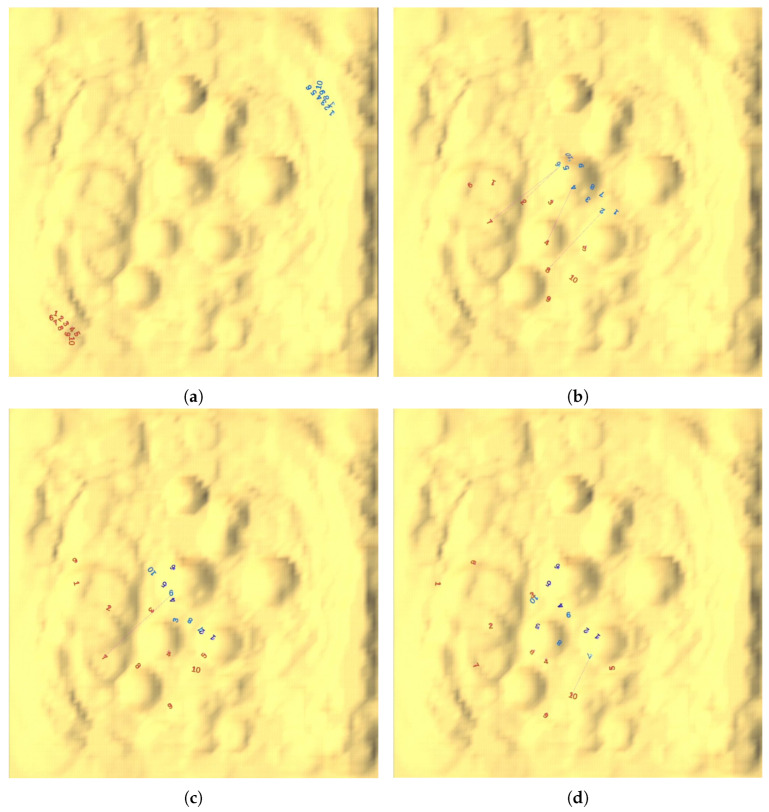

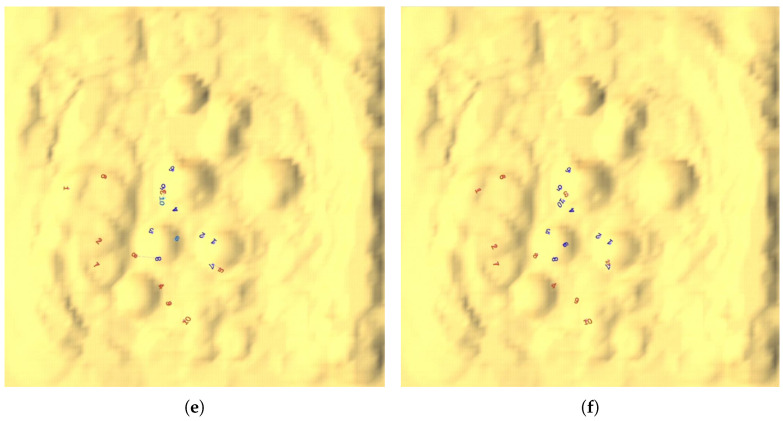
A diagram of the confrontation process. (**a**) Agents from both the red and blue teams are generated at diagonal positions on the map. (**b**) Both teams advance toward the higher hills on the map in search of opponents, while the red agents disperse in multiple directions. This maneuver leads to a direct confrontation between the two sides, prompting the red agents to initiate an attack. (**c**) After a round of attacks, the red agents begin to maneuver towards the rear. (**d**) The red agents form an encirclement around the opponent from multiple directions, employing strategies such as focused fire and flanking encirclement. (**e**) The red agents tighten the encirclement. (**f**) The match ends with all opponents eliminated, and the red team wins this round.

To more intuitively demonstrate the bio-inspired algorithm of agents during the confrontation process, this paper uses the AN algorithm as the opponent and selects a 10V10 confrontation scale for a detailed analysis of the confrontation process. The sequence of events is depicted in Figure 9.

In Figure 9d, blue agent $b_{8}$ becomes separated from the rest of its team during the confrontation, prompting red agents $r_{4}$, $r_{7}$, and $r_{8}$ to prioritize launching coordinated attacks on $b_{8}$. This process exemplifies the focused-fire strategy employed in the bio-inspired approach. Similarly, in Figure 9e, blue agent $b_{7}$ is also isolated, leading red agents $r_{5}$, $r_{9}$, and $r_{10}$ to direct their attacks toward it in accordance with the same focused-attack strategy.

In Figure 9a,b, without knowledge of the opponent’s positions, the red team disperses its formation in preparation for launching attacks from multiple directions. In Figure 9c–f, the red agents in different positions exhibit varying retreat directions, forming both a frontal containment and flanking maneuvers. Additionally, the red agents actively move to flank the opponent, as seen with agents $r_{5}$ and $r_{10}$ in Figure 9c,d, and agents $r_{4}$ and $r_{8}$ in Figure 9d,e. These coordinated attacks from different directions demonstrate the flanking encirclement strategy.

### 5.2. Analysis of Results Under Different Scenarios

The confrontation scenarios in this paper are constructed using the Unity platform, a widely adopted development tool in the electronic gaming industry. A total of 100 simulation tests are conducted against three opponents under varying algorithm parameters, confrontation scales, and map configurations to comprehensively evaluate the performance of the proposed algorithm. Before the confrontation begins, the environmental parameters are initialized with values of $v_{p}=1400\;m/s$, $t_{m}=200\;s$, $t_{cm}=2\;s$, $d_{mv}=1200\;m$, HP=3, $h_{d}=1$, v=20m/s, $w_{m}=25^{\circ }/\;s$, $\Omega_{m}=20^{\circ }/s$, $\Phi_{m}=15^{\circ }/s$, $d_{f}=100\;m$, $c_{t}=3$, $\theta_{s}=\pi /4$, $d_{a}=30\;m$, $\epsilon_{1}=30\;m$, $\epsilon_{2}=0.01^{\circ }$, $k_{1}=1$, and $k_{2}=0.5$. After each confrontation, the win rates and indices are recorded and analyzed to assess the impact of different parameter values on these outcomes.

#### 5.2.1. Analysis of Results Under Different Algorithm Parameters

The algorithm in this study includes two critical parameters, $d_{bc_{1}}$ and $d_{bc_{2}}$. Here, $d_{bc_{1}}$ represents the minimum distance for maneuvering and containment; if the distance between an agent and an opponent is less than $d_{bc_{1}}$, the agent will immediately maneuver backward. $d_{bc_{2}}$ represents the minimum distance for flanking during advancement and the maximum trigger distance for retreating and flanking maneuvers. When the distance between an agent and an opponent exceeds $d_{bc_{2}}$, agents on both sides will implement a flanking encirclement strategy. If the agent is in a firing cooldown state and the distance is less than $d_{bc_{2}}$, it will maneuver backward based on its position.

First, when both teams are in close proximity, agents may become overly clustered, leading to teammates obstructing the line of sight to opponents and diminishing the effectiveness of localized focused fire. This issue is further exacerbated when destroyed agents remain stationary at their last positions, increasing occlusion and reducing overall combat efficiency. To mitigate this, a minimum retreat distance threshold $d_{bc_{1}}$ is introduced to ensure adequate spacing between agents and opponents, thereby facilitating the execution of confrontation strategies. Second, since projectiles require a cooldown period after each attack, agents are temporarily unable to inflict damage on opponents. To enhance agent safety, a retreat trigger is activated based on the distance threshold $d_{bc_{2}}$, ensuring that agents maintain a safe distance from opponents while their attack systems are in cooldown. In summary, these two parameters play a crucial role in the proposed confrontation algorithm, balancing attack efficiency and spatial positioning to optimize engagement outcomes.

**Figure 10:**
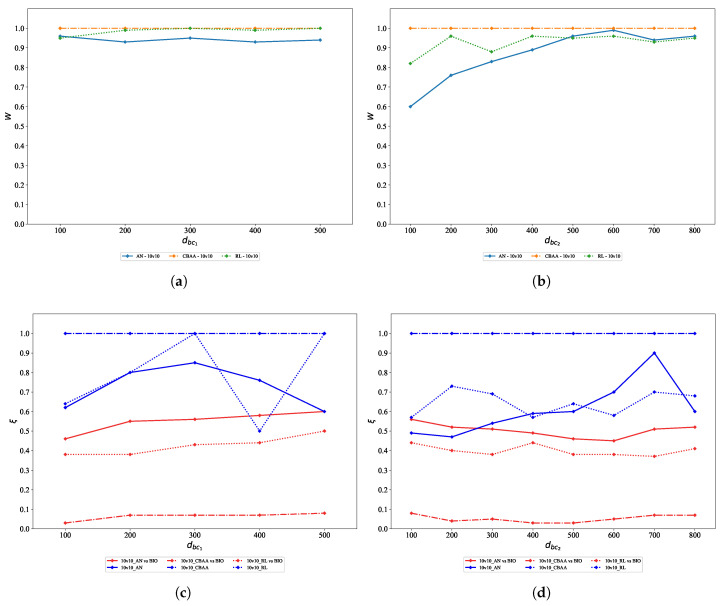

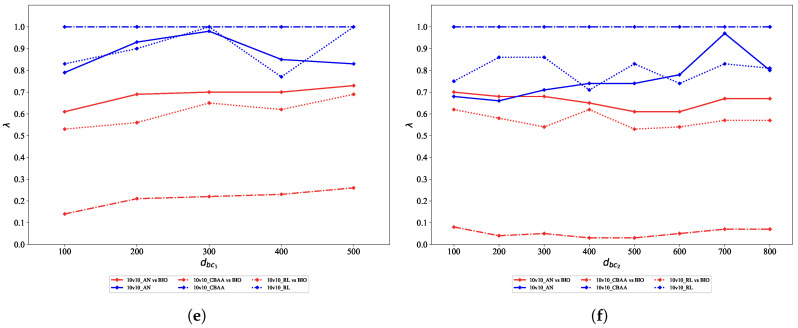
Upon fixing either parameter $d_{bc_{1}}$ or $d_{bc_{2}}$, and varying the other, the corresponding results are obtained. (**a**) Results of $W_{r}$ with varying $d_{bc_{1}}$. (**b**) Results of $W_{r}$ with varying $d_{bc_{2}}$. (**c**) Results of ξ with varying $d_{bc_{1}}$. (**d**) Results of ξ with varying $d_{bc_{2}}$. (**e**) Results of λ with varying $d_{bc_{1}}$. (**f**) Results of λ with varying $d_{bc_{2}}$.

When $d_{bc_{2}}=500\;m$, $d_{bc_{1}}$ is varied from 100m to 500m in increments of 100m. Conversely, when $d_{bc_{1}}=100\;m$, $d_{bc_{2}}$ is varied from 100m to 800m in increments of 100m. The confrontation win rates and indices for varying $d_{bc_{1}}$ are shown in Figure 10a–c. The win rates and indices for varying $d_{bc_{2}}$ are shown in Figure 10d–f.

First, we discuss the parameter $d_{bc_{1}}$. As shown in the figure, the algorithm’s win rate consistently exceeds 90%, indicating that variations in the value of $d_{bc_{1}}$ have little effect on the algorithm’s win rate. However, indices $\xi_{r}$ and $\lambda_{r}$ increase with the growth of $d_{bc_{1}}$, which indicates a decline in the algorithm’s performance. The agent’s backward maneuvering behavior is closely related to $d_{bc_{1}}$. When $d_{bc_{1}}=500\;m$, the agent retreats when it is still far from the opponent, leading to a more dispersed formation. Even if the agent is in a favorable attack position, it cannot quickly regroup to eliminate the opponent and may become isolated, resulting in concentrated enemy fire.

Next, we analyze $d_{bc_{2}}$. The win rate graph shows that $d_{bc_{2}}$ significantly impacts the algorithm’s performance. This is primarily due to the fast shell speed of the agents—when $d_{bc_{2}}$ is set to a smaller value, agents are more likely to engage in direct encounters, with a high probability of being hit. A smaller $d_{bc_{2}}$ also leads to prolonged exposure in the line of sight, limiting the ability to fully leverage terrain for tactical maneuvering. As a result, the attack pattern often degenerates into direct firefights. However, as $d_{bc_{2}}$ increases, the win rate improves. For example, against AN, the win rate $W_{r}$ increases from 0.60 at $d_{bc_{2}}=100\;m$ to 0.96 at $d_{bc_{2}}=500\;m$. Both $\xi_{r}$ and $\lambda_{r}$ initially decrease as $d_{bc_{2}}$ rises within the range [100, 600] m, but increase again when $d_{bc_{2}}>600\;m$. For instance, when facing AN, as $d_{bc_{2}}$ increases from 100 m to 600 m, $\xi_{r}$ decreases from 0.56 to 0.45, while $\lambda_{r}$ drops from 0.70 to 0.61. When an agent is positioned closer to an opponent, the duration of direct engagement increases, reducing the agent’s maneuverability and making it more susceptible to concentrated opponent attacks. An agent will initiate a retreat only when its distance to the opponent falls below $d_{bc_{2}}$ and its attack system is in a cooldown state. When $d_{bc_{2}}$ is relatively large, the retreat trigger zone falls within the interval $\left[d_{bc_{1}},d_{bc_{2}}\right]$, which may cause the team to become overly dispersed, thereby weakening the effectiveness of the flanking encirclement strategy. Although retreat-oriented behavior can help maintain a high win rate, agents become more likely to be targeted and defeated through focused opponent attacks, ultimately degrading the algorithm’s overall performance.

#### 5.2.2. Analysis of Results Under Different Confrontation Scales

**Figure 11:**
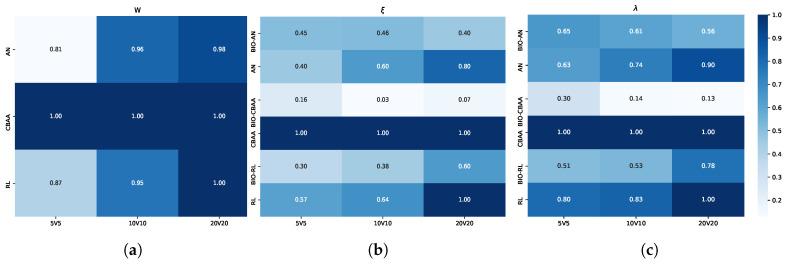
Results under different confrontation scales. (**a**) Results of $W_{r}$ under different confrontation scales. (**b**) Results of ξ under different confrontation scales. (**c**) Results of λ under different confrontation scales.

The results under different confrontation scales are shown in Figure 11. From the confrontations at different scales, it is evident that larger confrontation scales lead to higher win rates for the algorithm, a trend especially pronounced when the opponent is AN. In 5v5 scenarios, the total health of the team is relatively lower compared to larger scales, and fewer agents are involved in flanking and localized focused-fire maneuvers. As a result, even when a flanking formation is established, if an agent on one side encounters the opponent head-on and is in a disadvantageous position, it may be quickly eliminated, causing the flanking encirclement strategy to collapse. Consequently, the win rate in such cases is only 0.81. However, as the scale increases, the bio-inspired strategy allows for a more complete formation. The increase in the number of agents on each side improves the margin for error, provides more firing points, and enables the agents to eliminate targets more quickly. On a scale of 20v20, the win rate consistently exceeds 95%.

The indices of the algorithm also vary with the confrontation scale. When facing AN and CBAA, the algorithm’s indices improve as the confrontation scale increases. Both algorithms are based on target selection, making the flanking encirclement strategy proposed in this paper highly effective. An increased confrontation scale leads to a greater number of attack positions and dilutes the opponent’s offensive intensity, thereby accelerating the elimination of opponents and mitigating team losses. From 5v5 to 20v20, both $\xi_{r}$ and $\lambda_{r}$ drop by over 10. However, when facing RL, the results for $\xi_{r}$ and $\lambda_{r}$ increase by more than 30% from 5v5 to 20v20. This is because the RL algorithm defaults to targeting the nearest opponent, and once an attack target is locked, agents using RL tend to charge aggressively. Suppose agents equipped with the BIO algorithm fail to form a proper formation in time. This results in clustering, increasing agent and HP losses, thereby reducing the algorithm’s overall performance.

#### 5.2.3. Result Analysis on Different Maps

**Figure 12:**
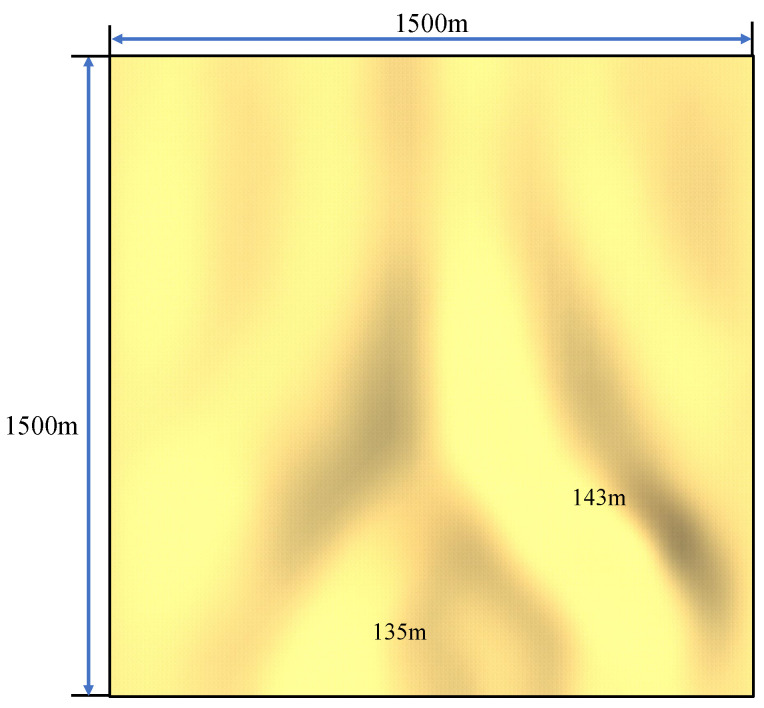
A hilltop panoramic view of the other hilly terrain. This map shares the same dimensions as the first one but exhibits steeper overall slopes with less pronounced elevation variations.

**Figure 13:**
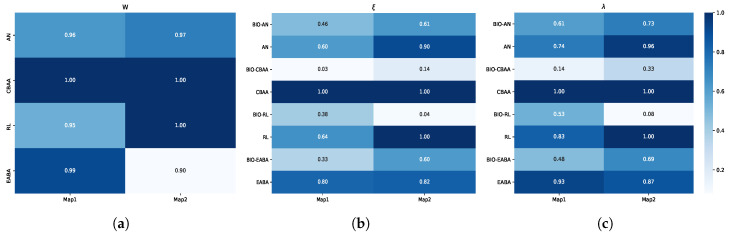
Results using different confrontation maps. (**a**) Results of $W_{r}$ under different confrontation maps. (**b**) Results of ξ under different confrontation maps. (**c**) Results of λ under different confrontation maps.

In addition to the current confrontation map, we conducted tests on another map. Compared to the previous map, this one has a gentler slope, and the specific terrain is shown in Figure 12. Furthermore, an additional comparative algorithm, the Evolutionary Algorithm-Based Attack (EABA) Strategy [16], was introduced in the other map. The confrontation scale was 10v10, with $d_{bc_{1}}=100\;m$ and $d_{bc_{2}}=500\;m$. The confrontation results are shown in Figure 13.

From the results, it can be observed that the win rate of the algorithm in this paper remains above 90%. When facing AN and CBAA opponents, both $\xi_{r}$ and $\lambda_{r}$ show slight increases. For example, in the confrontation against AN, $\xi_{r}$ increases from 0.46 to 0.61, and $\lambda_{r}$ rises from 0.61 to 0.73. Due to the flatter terrain, the probability of shells being obstructed by the ground during flight is lower, which increases the likelihood that red team agents may be hit by opponent shells while spreading out to form a flanking formation, resulting in higher losses for their team. Conversely, when facing RL, both $\xi_{r}$ and $\lambda_{r}$ show a slight decrease, which can be attributed to the RL model’s weaker adaptability to the new map, leading to lower confrontation performance. When confronting the EABA algorithm, the proposed approach yields a lower $w_{r}$, while both performance indices, $\xi_{r}$ and $\lambda_{r}$, show noticeable increases. This phenomenon primarily results from the flatter terrain, which improves the likelihood of acquiring opponent position information. With enhanced visibility, the EABA algorithm can better exploit its fitness function through iterative optimization, thereby strengthening its confrontation capabilities and negatively impacting the performance of the proposed algorithm. In summary, this paper’s algorithm maintains a high win rate on the new confrontation map and achieves better $\xi_{r}$ and $\lambda_{r}$ results compared to its opponents, demonstrating the algorithm’s advantages in different environments.

## 6. Conclusions

From the perspective of electronic game scenarios, this paper explores a swarm confrontation algorithm designed for complex hilly terrains. A highly dynamic hilly confrontation environment is constructed, where intelligent agent swarms from both a red and a blue team possess equal numbers and identical capabilities, with each agent’s movements constrained by kinematic limitations. Drawing inspiration from the hunting confrontation behaviors of wild dog packs and lion prides in nature, two key strategies are proposed: a focused-fire strategy for target selection and a flanking encirclement strategy for motion planning. The former improves local performance by aggregating agent behaviors toward a shared objective, while the latter improves overall confrontation efficiency through coordinated movement and poditioning. To comprehensively evaluate the algorithm’s performance, the proposed approach is benchmarked against three existing confrontation algorithms. A total of 100 confrontation tests are conducted across different algorithm parameters, confrontation scales, and environmental conditions. The experimental results demonstrate that the proposed algorithm achieves a confrontation win rate exceeding 80% against baseline algorithms while maintaining lower average agent loss rates and a reduced average agent health loss rate. In conclusion, this biologically inspired confrontation algorithm not only offers a straightforward and practical solution but also exhibits superior performance in swarm-based confrontations.

For future work, we suggest an in-depth exploration of opponent searching in environments with denied information to enhance the algorithm’s confrontation capabilities under limited visibility. Additionally, examining the impact of communication constraints, such as delays and packet loss, on swarm coordination and overall performance will be essential. Developing robust algorithms to mitigate these challenges will be a key focus moving forward.

## Data Availability

The data of this paper are available upon request.

## Nomenclature

*N*	The total number of agents in each team
$r_{i}$	The *i*th agent of the red team
$b_{i}$	The *i*th agent of the blue team
$p_{ri}\left(t\right)$	The position of agent $r_{i}$
*v*	The linear speed
$\omega_{ri}\left(t\right)$	The angular velocity of agent $r_{i}$
$\theta_{ri}\left(t\right)$	The heading angle of agent $r_{i}$
$\varphi_{ri}\left(t\right)$	The pitch angle of agent $r_{i}$
$\vartheta_{ri}\left(t\right)$	The heading angle of agent $r_{i}$’s turret
$\Omega_{ri}\left(t\right)$	The rotation speed of agent $r_{i}$’s turret
$\sigma_{ri}\left(t\right)$	The heading angle of agent $r_{i}$’s barrel
$\Phi_{ri}\left(t\right)$	The rotation speed of agent $r_{i}$’s barrel
Δt	The sampling time
$d_{mv}$	The maximum detection range of the agents
$N_{ri}\left(t\right)$	The set of opponents whose information can be obtained by agent $r_{i}$ at time *t*
$\omega_{m}$	The maximum speed of $\omega_{ri}\left(t\right)$
$\Omega_{m}$	The maximum speed of $\Omega_{ri}\left(t\right)$
$\Phi_{m}$	The maximum speed of $\Phi_{ri}\left(t\right)$
$v_{p}$	The initial speed of the shells
$h_{d}$	The damage inflicted on an agent upon being hit by a single shell
$h_{ri}\left(t\right)$	The health point of $r_{i}$ at time *t*
$t_{m}$	The maximum execution time per confrontation
*M*	The total number of matches played between the red and blue teams
$M_{w}^{r}$	The total number of matches won by the red team
$H_{s}^{r}$	The initial health points of all members of the red team
$n_{k}^{r}$	The number of agents lost by the red team in the *k*th winning match
$h_{sk}^{r}$	The total health points lost by the red team in the *k*th winning match
$W_{r}$	The winning rate of $r_{i}$
$\xi_{r}$	The average agent quantity loss rate of the red team
$\lambda_{r}$	The average agent health loss rate of the red team
$n_{b_{a}}^{r_{i}}\left(t\right)$	The number of surviving opponents detectable by $r_{i}$
$n_{b_{a}}\left(t\right)$	The number of surviving agents on the blue team
$p_{c_{b}}^{r_{i}}\left(t\right)$	The central position of these opponents
$I_{x}^{r_{i}}\left(t\right)$	The label of the *x*th closest surviving opponent to $r_{i}$
$T_{ri}\left(t\right)$	The final attack target selected by $r_{i}$
$p_{b_{l}}^{r_{i}}\left(t\right)$	The position of $r_{i}$’s current attack target
$p_{m}^{ri}\left(t\right)$	The position of the nearest hilltop to $r_{i}$
$G_{ri}\left(t\right)$	The movement direction of $r_{i}$ without obstacle avoidance
$p_{b_{T}}^{r_{i}}\left(t\right)$	The position of the opponent labeled $T_{ri}$
$X_{k}^{r_{i}}\left(t\right)$	The obstacle avoidance vector induced by teammate $r_{k}$ on $r_{i}$
$X_{ri}\left(t\right)$	The sum of obstacle avoidance vectors exerted by all teammates on $r_{i}$
$F_{ri}\left(t\right)$	The final desired movement direction of $r_{i}$
$\rho_{r_{i}}\left(t\right)$	The relative position of $r_{i}$ within the friendly team that shares the same opponent
$p_{c}^{r_{i}}\left(t\right)$	The position of the agent closest to $p_{b_{T}}^{r_{i}}\left(t\right)$ among the group of agents sharing the same attack target
$l_{ri}$	The unit direction vector from $p_{c}^{r_{i}}\left(t\right)$ to $p_{b_{o}}^{r_{i}}\left(t\right)$
$l_{z}$	The unit direction vector along the *z*-axis
$d_{i}^{r}\left(t\right)$	The projected offset within a team sharing the same attack target
$\epsilon_{1}$	The reference value used to determine the position interval
$d_{r_{k}}^{r_{i}}\left(t\right)$	The distance between $r_{i}$ and $r_{k}$
$d_{b_{j}}^{r_{i}}\left(t\right)$	The distance between $r_{i}$ and $b_{j}$
$t_{c}^{r_{i}}$	The time elapsed since $r_{i}$ fired its last shell
$t_{cm}$	The minimum firing interval between two attacks
$d_{bc_{1}}$	The maximum distance threshold for $r_{i}$ to execute a retreating flanking encirclement strategy
$d_{bc_{2}}$	The minimum distance threshold for $r_{i}$ to execute a flanking maneuver during an advance, as well as the minimum retreat distance for flanking when $t_{c}^{r_{i}}<t_{cm}$
$d_{a}$	The distance for avoiding teammates
$\theta_{F}^{r_{i}}\left(t\right)$	The heading angle of $F_{ri}\left(t\right)$
$e_{\theta 1}^{r_{i}}\left(t\right)$	The clockwise angle between the current movement direction and the final target direction
$e_{\theta 2}^{r_{i}}\left(t\right)$	The counterclockwise angle between the current movement direction and the final target direction
$u_{tur}^{r_{i}}\left(t\right)$	The unit direction vector of $r_{i}$’s turret
$u_{bar}^{r_{i}}\left(t\right)$	The unit direction vector of $r_{i}$’s barrel
$f_{a}^{r_{i}}\left(t\right)$	The flag indicating whether $r_{i}$ is actively aiming at an opponent
$u_{o}^{r_{i}}\left(t\right)$	The unit vector from $r_{i}$ to $T_{ri}\left(t\right)$
$u_{o1}^{r_{i}}\left(t\right)$	The projection of $u_{o}^{r_{i}}\left(t\right)$ onto the XOY plane
$u_{tur1}^{r_{i}}\left(t\right)$	The projection of $u_{tur}^{r_{i}}\left(t\right)$ onto the XOY plane
$\theta_{tur}^{r_{i}}\left(t\right)$	The angle formed between $u_{o1}^{r_{i}}\left(t\right)$ and $u_{tur1}^{r_{i}}\left(t\right)$
$\theta_{bar}^{r_{i}}\left(t\right)$	The angle formed between $u_{o}^{r_{i}}\left(t\right)$ and $u_{bar}^{r_{i}}\left(t\right)$
$\epsilon_{2}$	The deviation range between the target angle and the actual angle

## References

[1] Ayamga M., Akaba S., Nyaaba A.A. (2021). Multifaceted applicability of drones: A review. Technol. Forecast. Soc. Change.

[2] Day M. (2012). Multi-Agent Task Negotiation Among UAVs to Defend Against Swarm Attacks. Ph.D. Thesis.

[3] Kong L., Liu Z., Pang L., Zhang K., Long S., Dhillon B.S. (2023). Research on UAV Swarm Operations. Man-Machine-Environment System Engineering.

[4] Niu W., Huang J., Miu L. (2018). Research on the concept and key technologies of unmanned aerial vehicle swarm concerning naval attack. Command. Control. Simul..

[5] Xiaoning Z. Analysis of military application of UAV swarm technology. Proceedings of the IEEE 2020 3rd International Conference on Unmanned Systems (ICUS).

[6] Vinyals O., Babuschkin I., Czarnecki W.M., Mathieu M., Dudzik A., Chung J., Choi D.H., Powell R., Ewalds T., Georgiev P. (2019). Grandmaster level in StarCraft II using multi-agent reinforcement learning. Nature.

[7] Xia W., Zhou Z., Jiang W., Zhang Y. (2023). Dynamic UAV Swarm Confrontation: An Imitation Based on Mobile Adaptive Networks. IEEE Trans. Aerosp. Electron. Syst..

[8] Zhang L., Yu X., Zhang S. Research on Collaborative and Confrontation of UAV Swarms Based on SAC-OD Rules. Proceedings of the 4th International Conference on Information Management and Management Science. Association for Computing Machinery.

[9] Raslan H., Schwartz H., Givigi S., Yang P. (2016). A Learning Invader for the “Guarding a Territory” Game. J. Intelligent Robot. Syst..

[10] Yang X.S., Deb S., Zhao Y.X., Fong S., He X. (2018). Swarm intelligence: Past, present and future. Soft Comput..

[11] Zervoudakis K., Tsafarakis S. (2020). A mayfly optimization algorithm. Comput. Ind. Eng..

[12] Połap D., Woźniak M. (2021). Red fox optimization algorithm. Expert Syst. Appl..

[13] Zervoudakis K., Tsafarakis S. (2023). A global optimizer inspired from the survival strategies of flying foxes. Eng. Comput..

[14] Li J., Yang S.X. (2024). Intelligent Fish-Inspired Foraging of Swarm Robots with Sub-Group Behaviors Based on Neurodynamic Models. Biomimetics.

[15] Chi P., Wei J., Wu K., Di B., Wang Y. (2023). A Bio-Inspired Decision-Making Method of UAV Swarm for Attack-Defense Confrontation via Multi-Agent Reinforcement Learning. Biomimetics.

[16] Liu H., Zhang J., Zu P., Zhou M. (2023). Evolutionary Algorithm-Based Attack Strategy With Swarm Robots in Denied Environments. IEEE Trans. Evol. Comput..

[17] Wang Y., Bai P., Liang X., Wang W., Zhang J., Fu Q. (2019). Reconnaissance Mission Conducted by UAV Swarms Based on Distributed PSO Path Planning Algorithms. IEEE Access.

[18] Yu Y., Liu J., Wei C. (2022). Hawk and pigeon’s intelligence for UAV swarm dynamic combat game via competitive learning pigeon-inspired optimization. Sci. China Technol. Sci..

[19] Zhang T., Chai L., Wang S., Jin J., Liu X., Song A., Lan Y. (2022). Improving Autonomous Behavior Strategy Learning in an Unmanned Swarm System Through Knowledge Enhancement. IEEE Trans. Reliab..

[20] Xiang L., Xie T. Research on UAV Swarm Confrontation Task Based on MADDPG Algorithm. Proceedings of the 2020 5th International Conference on Mechanical, Control and Computer Engineering (ICMCCE).

[21] Finand B., Loeuille N., Bocquet C., Fédérici P., Monnin T. (2024). Solitary foundation or colony fission in ants: An intraspecific study shows that worker presence and number increase colony foundation success. Oecologia.

[22] Liu F., Dong X., Yu J., Hua Y., Li Q., Ren Z. (2022). Distributed Nash Equilibrium Seeking of *N*-Coalition Noncooperative Games With Application to UAV Swarms. IEEE Trans. Netw. Sci. Eng..

[23] Guo Z., Li Y., Wang Y., Wang L. (2023). Group motion control for UAV swarm confrontation using distributed dynamic target assignment. Aerosp. Syst..

[24] Wang L., Qiu T., Pu Z., Yi J., Zhu J., Zhao Y. (2023). A Decision-making Method for Swarm Agents in Attack-defense Confrontation. IFAC-PapersOnLine.

[25] Xing D., Zhen Z., Gong H. (2019). Offense-defense confrontation decision making for dynamic UAV swarm versus UAV swarm. Proc. Inst. Mech. Eng. Part G J. Aerosp. Eng..

[26] Gergal E.K. (2021). Drone Swarming Tactics Using Reinforcement Learning and Policy Optimization.

[27] Cai H., Luo Y., Gao H., Chi J., Wang S. (2023). A Multiphase Semistatic Training Method for Swarm Confrontation Using Multiagent Deep Reinforcement Learning. Comput. Intell. Neurosci..

[28] Wang B., Li S., Gao X., Xie T. (2021). UAV Swarm Confrontation Using Hierarchical Multiagent Reinforcement Learning. Int. J. Aerosp. Eng..

[29] Shahid S., Zhen Z., Javaid U., Wen L. (2022). Offense-Defense Distributed Decision Making for Swarm vs. Swarm Confrontation While Attacking the Aircraft Carriers. Drones.

[30] Wang J., Duan S., Ju S., Lu S., Jin Y. (2022). Evolutionary Task Allocation and Cooperative Control of Unmanned Aerial Vehicles in Air Combat Applications. Robotics.

[31] Xu C., Xu M., Yin C. (2020). Optimized multi-UAV cooperative path planning under the complex confrontation environment. Comput. Commun..

[32] Xuan W., Weijia W., Kepu S., Minwen W. (2019). UAV Air Combat Decision Based on Evolutionary Expert System Tree. Ordnance Ind. Autom..

[33] Sun B., Zeng Y., Zhu D. (2024). Dynamic task allocation in multi autonomous underwater vehicle confrontational games with multi-objective evaluation model and particle swarm optimization algorithm. Appl. Soft Comput..

[34] Hu S., Ru L., Lv M., Wang Z., Lu B., Wang W. (2024). Evolutionary game analysis of behaviour strategy for UAV swarm in communication-constrained environments. IET Control. Theory Appl..

[35] Shefaei A., Mohammadi-Ivatloo B. (2018). Wild Goats Algorithm: An Evolutionary Algorithm to Solve the Real-World Optimization Problems. IEEE Trans. Ind. Inform..

[36] Wu M., Zhu X., Ma L., Wang J., Bao W., Li W., Fan Z. (2022). Torch: Strategy evolution in swarm robots using heterogeneous–homogeneous coevolution method. J. Ind. Inf. Integr..

[37] Bai Z., Zhou H., Shi J., Xing L., Wang J. (2024). A hybrid multi-objective evolutionary algorithm with high solving efficiency for UAV defense programming. Swarm Evol. Comput..

[38] Xu D., Chen G. (2022). Autonomous and cooperative control of UAV cluster with multi-agent reinforcement learning. Aeronaut. J..

[39] Xu D., Chen G. (2022). The research on intelligent cooperative combat of UAV cluster with multi-agent reinforcement learning. Aerosp. Syst..

[40] Nian X., Li M., Wang H., Gong Y., Xiong H. (2024). Large-scale UAV swarm confrontation based on hierarchical attention actor-critic algorithm. Appl. Intell..

[41] Kong W., Zhou D., Yang Z., Zhang K., Zeng L. (2020). Maneuver Strategy Generation of UCAV for within Visual Range Air Combat Based on Multi-Agent Reinforcement Learning and Target Position Prediction. Appl. Sci..

[42] Zhou K., Wei R., Zhang Q., Wu Z. Research on Decision-making Method for Territorial Defense Based on Fuzzy Reinforcement Learnin. Proceedings of the 2019 Chinese Automation Congress (CAC).

[43] Jiang F., Xu M., Li Y., Cui H., Wang R. (2023). Short-range air combat maneuver decision of UAV swarm based on multi-agent transformer introducing virtual objects. Eng. Appl. Artif. Intell..

[44] Wang Z., Liu F., Guo J., Hong C., Chen M., Wang E., Zhao Y. UAV Swarm Confrontation Based on Multi-agent Deep Reinforcement Learning. Proceedings of the 2022 41st Chinese Control Conference (CCC).

[45] Fang J., Han Y., Zhou Z., Chen S., Sheng S. (2021). The collaborative combat of heterogeneous multi-UAVs based on MARL. J. Phys. Conf. Ser..

[46] Kouzeghar M., Song Y., Meghjani M., Bouffanais R. Multi-Target Pursuit by a Decentralized Heterogeneous UAV Swarm using Deep Multi-Agent Reinforcement Learning. Proceedings of the 2023 IEEE International Conference on Robotics and Automation (ICRA).

[47] Youtube Wild Dogs vs. Wildebeests. https://www.youtube.com/watch?v=h4SlAc2U1A4.

[48] Youtube Lions vs. Buffalo—A Wild Encounter. https://www.youtube.com/watch?v=t7KMsIdlx1E.

